# Correlation of Palatal Index With Pharyngeal Airway in Various Skeletal Patterns

**DOI:** 10.7759/cureus.39032

**Published:** 2023-05-15

**Authors:** Yasaswini Aluru, Rajesh RNG, Anadha N Gujar, Rony Kondody

**Affiliations:** 1 Orthodontics and Dentofacial Orthopaedics, Sri Rajiv Gandhi College of Dental Sciences, Bengaluru, IND; 2 Orthodontics and Dentofacial Orthopaedics, Sri Rajiv Gandhi College of Dental Sciences and Hospital, Bengaluru, IND

**Keywords:** upper airway, palatal index, lower airway, cephalometry, airway

## Abstract

Introduction

This retrospective study aimed to correlate palatal index with pharyngeal airway in class I, class II and class III skeletal patterns.

Materials and methods

A total of 30 individuals with a mean age of 17.5 years were included in the study. The subjects were categorized on the basis of ANB (A point, nasion, B point) angle into skeletal class I, II, and III patterns (N=10). Using Korkhaus analysis, palatal height, palatal breadth, and palatal height index were calculated from the study models. From the lateral cephalogram, the dimensions of the upper and lower pharyngeal airways were measured using McNamara Airway Analysis. The results were calculated using the ANOVA test.

Results

A statistically significant difference was found in all three groups of class I, II, and III malocclusions for palatal index and airway dimensions. The skeletal class II malocclusion participants exhibited the highest mean values for the palatal index (P=0.03). Class I had the highest mean value for the upper airway (P=0.041), whereas class III had the highest mean value for the lower airway (P=0.026).

Conclusion

It was concluded that subjects with the class II skeletal pattern have a high palate and reduced upper and lower airways when compared with class I and class III skeletal patterns, which showed larger upper and lower airways, respectively.

## Introduction

In the field of orthodontics, the unique traits and structural relationships of the palate depth, width, and airway dimensions are crucial in determining a person’s facial structure, thus helping in the diagnosis of malocclusions. Over the past few decades, interest in upper and lower airway dimensions has progressively increased, mostly because of an understanding of the connection between upper airway configuration and sleep-disordered breathing [1] and its connection with craniofacial morphology, in general.

Numerous studies have been done on the palate; some of them aimed to trace the median sagittal and transverse contours of the palate at different developmental stages in dental casts to investigate changes in the growth of the palate [2]. A high or narrow palate can be a feature of some disorders, including Turner’s syndrome, Apert’s syndrome, Treacher-Collin syndrome, etc. [3].

Numerous variables affect how occlusal patterns and craniofacial shape develop. A detailed investigation needs to be conducted into upper airway obstruction and how it affects dental development and craniofacial growth. Mouth breathing has been associated with the emergence of skeletal and dental anomalies, according to clinical investigations [4,5].

Upper airway alterations in orthodontics must always be assessed clinically at the beginning of the treatment, as well as by lateral cephalograms or cone beam computed tomography (CBCT)*. *The information offered by cephalometry is sparse since it reconstructs three-dimensional features into two-dimensional ones. On the other hand, CBCT provides a lot of diagnostic data because it displays three-dimensional structures, allowing us to quantify the volume of various structures; moreover, it also constructs projections on several planes.

Obstruction of the upper airways makes breathing even more difficult and can cause malocclusion, jaw deformity, and craniofacial abnormality. Additionally, studies have shown that abnormal craniofacial development can cause a lifetime of health problems like airway blockage, impaired respiration, impaired nasal breathing, chronic mouth breathing, sleep apnea, and sleep disturbances [6]. Both the craniofacial form and function may come first in the craniofacial hierarchy. So, orthodontic and orthopedic therapy should be used to carefully manage both the craniofacial shape and function, especially during the early stages of growth and development.

The palatal height index is obtained from the combination of palatal width and palatal depth which was first introduced by Korkhaus [4]. The upper and lower pharyngeal airway measurements are taken from the McNamara airway analysis [7]

A study should be conducted to analyze airway and palatal morphology in depth for better treatment-plan formulation because there appears to be heterogeneity in the shape of palatal vaults in each skeletal pattern. Moreover, airway and craniofacial development are also interrelated. Therefore, the aim of our study is to correlate palatal index with pharyngeal airway in class I, II, and III skeletal patterns.

## Materials and methods

The study was carried out at the Department of Orthodontics and Dentofacial Orthopaedics of Sri Rajiv Gandhi Dental Hospital, Bengaluru, India. Lateral cephalograms and dental stone models of 30 subjects were randomly selected from the patients who visited the department seeking orthodontic treatment. The sample size was calculated by using G*Power software version 3.1.9.2(Erdfelder, Faul, & Buchner, Germany). The subjects were divided into three groups - Class I, II, and III - based on the type of sagittal relationship using the ANB (A point, nasion, B point) angle and wits appraisal.

Group I included patients with class I skeletal bases with ANB values in the range of 0° to 2°. Group II had patients with class II skeletal bases with ANB values greater than 2°. Finally, group III patients had class III skeletal bases with ANB value of less than 0°.

The lateral cephalograms were obtained in a natural head posture, in which the subjects looked at the reflection of their eyes in a mirror placed 5 ft in front of them and the teeth were in centric occlusion with the Frankfort horizontal plane parallel to the floor. The position was stabilized with ear rods and nasal support to prevent the head from rotating during exposure.

All the cephalogram tracings were done manually on clear acetate sheets using a 2H pencil, and the upper and lower airway dimensions were recorded based on McNamara airway analysis for all the patients [7].

**Figure 1:**
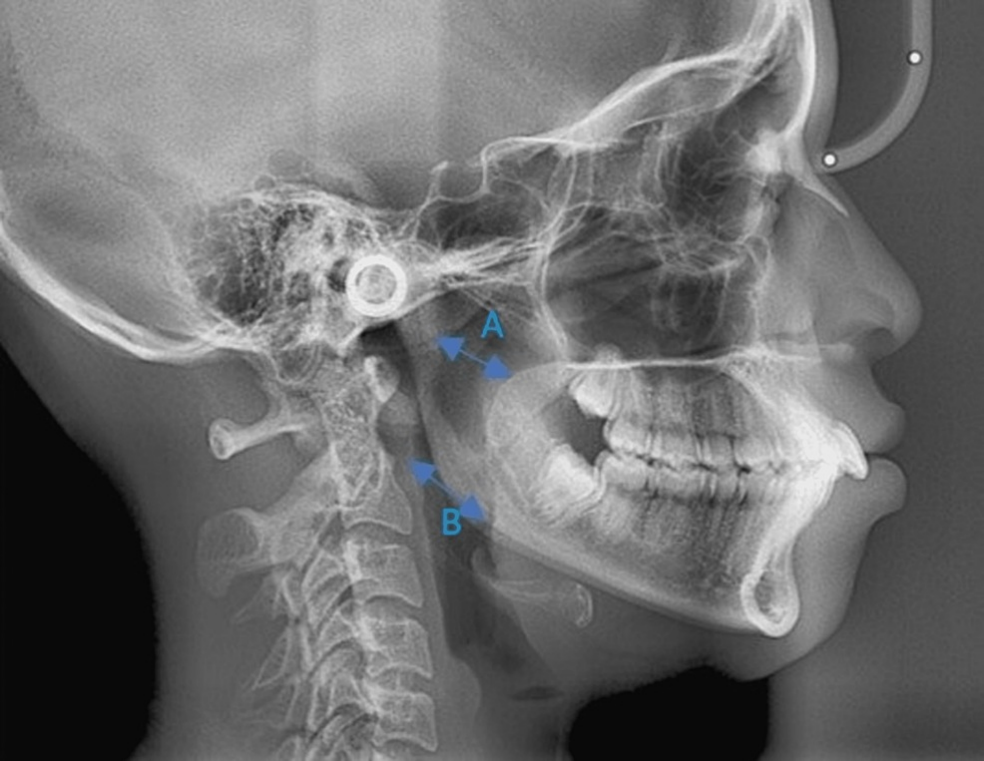
Measurement of the upper airway (A) and lower airway (B) on a lateral cephalogram

The upper pharyngeal width is measured from a point on the posterior outline of the soft palate to the closest point on the posterior pharyngeal wall. This measurement is taken on the anterior half of the soft palate outline because the area immediately adjacent to the posterior opening of the nose is critical in determining upper respiratory patency. The head film outline of the nasopharynx is a two-dimensional representation of a three-dimensional structure (Figure 1). The lower pharyngeal width is measured from the intersection of the posterior border of the tongue and the inferior border of the mandible to the closest point on the posterior pharyngeal wall (Figure 1) [8].

The impressions for obtaining the study models were made with alginate impression material (Algitex, DPI company, Mumbai, India), and the impressions and the study models are made with Type 3 Gypsum (Orthokal, Kalabhai Karson Pvt. Ltd, Chennai, India).

In the Korkhaus analysis, the palatal index was individually calculated on the study models for all the subjects using a divider and scale. The width was the distance between the maxillary first permanent molars at the cervical line. The height was the shortest distance between the midline of the junction of the hard and soft palates and the plane established by the other reference points [4].

**Figure 2:**
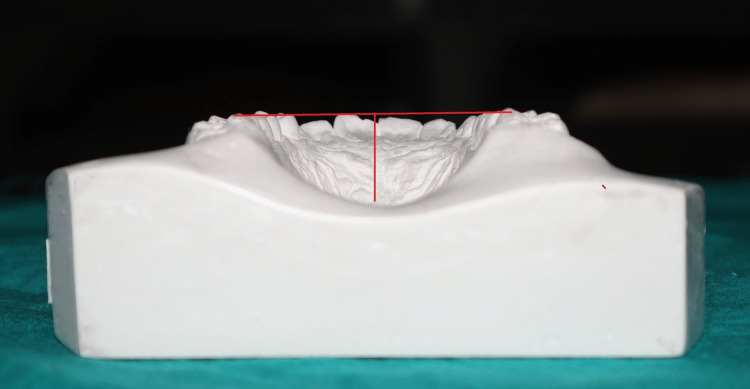
Study model showing palatal height and width

The palatal height Index was calculated using the following formula (Figure 2): Palatal height index = palatal height/palatal width × 100

Statistical analysis

Data analysis was done using IBM SPSS software for Windows, version 21 (IBM Corp., Armonk, USA). The ANOVA test was used to determine the difference between the three classes in terms of the palatal index, lower airway, and upper airway. Descriptive statistics were used to determine the standard deviation and the mean of the palatal index, upper airway, and lower airway in class I, II, and III.

## Results

The descriptive statistics and comparison of the palatal index with the upper and lower airway using the ANOVA test are shown in Tables 1-3. Table 1 depicts the mean and standard deviations of the palatal index for all three classes and the results of the ANOVA test which shows that there is a statistically significant difference between the three classes in terms of the palatal index and class II has the high mean value (47.20).

**Table 1:** ANOVA test for palatal index

Palatal index					
Class	N	Mean	Standard deviation	F-value	P-value
Class I	10	43.720	1.726	1.777	0.03^*^
Class II	10	47.200	5.788		
Class III	10	43.040	6.065		

**Table 2:** ANOVA test for upper airway

Upper airway					
Class	N	Mean	Standard deviation	F-value	P-value
Class I	10	11.90	3.315	0.174	0.041^*^
Class II	10	11.80	2.486		
Class III	10	11.10	2.751		

**Table 3:** ANOVA test for lower airway

Lower airway					
Class	N	Mean	Standard deviation	F-value	P-value
Class I	10	10.20	2.860	2.562	0.026^*^
Class II	10	10.00	2.789		
Class III	10	12.90	3.843		

Table 2 shows that the mean upper airway was greatest in class I (11.90) among the three classes and a p-value of 0.04 for the ANOVA test infers that there exists a difference between the three classes and it was statistically significant.

Table 3 shows that the mean lower airway was highest in class III (12.90) and the difference between the three classes in terms of the lower airway was statistically significant with a p-value of 0.026.

## Discussion

Ethnicity, dietary practices, and environmental factors have all been reported to have an impact on palatal dimensions. Every racial or racial affinity has a distinctive facial and cranial structure. People from different cultures and countries may also differ from each other in terms of their traits and facial features [8]. Additionally, the craniofacial complex includes the face, so the key indicator of the anatomical structure that can distort the skeletal pattern is the palate morphology [9]. Thus, having an understanding of the morphometrics of the hard palate is undoubtedly helpful in a variety of dental specialties, including orthodontics and orthognathic surgery [10,11].

Since airway and craniofacial development are interrelated, our study aimed to correlate palatal depth with pharyngeal airway in class I, II, and III malocclusions.

In this study, the mean and standard deviations of the palatal index for all three classes were calculated and compared using the ANOVA test. The results showed that there is a statistically significant difference between all three classes in terms of the palatal index, and class II has the highest mean value. This suggests that, in subjects with class II malocclusion, the palatal index was higher than that found in average subjects. This could be because class II malocclusion comprises a wide variety of arch-form deviations and a diverse etiology behind it, including a habit of sucking thumb. Thumb sucking is a primary cause of high palatal vaults [12]. This was found in the studies conducted by Linder A [13], Gwynne-Evans [14], and Klein [15]. They found that palatal height increased in mouth breathers, patients with adenoid hypertrophy, and patients with a habit of sucking thumb. Those cases also showed a class II malocclusion.

This study shows that the mean upper airway was the greatest in class I patients among the three classes, depicting a statistically significant difference between the three classes. Class II malocclusions are a result of the tongue being positioned backward, in accordance with Balter's hypothesis[16]. Incorrect deglutition and mouth breathing result from the obstruction of the respiratory function in the pharynx region. So, class I malocclusion had a higher mean value compared with other malocclusions. This was in accordance with a study conducted by Jain et al. [17]. Another study conducted by Flores-Blancas et al. [18] reported similar results: brachyfacial individuals with class I malocclusion have wider nasopharyngeal linear anteroposterior widths than mesofacial and dolichofacial people.

In this study, the mean lower airway was highest in class III patients, and the difference between the three classes in terms of the lower airway was statistically significant. This suggests that all the lower pharyngeal airway characteristics had considerably higher values in skeletal class III malocclusion samples than in those of skeletal class I and II samples. This could be a sign of a forward tongue posture, which may be one of the causes of skeletal class III malocclusion. The results found by McNamara [7], who proposed that a forward-placed tongue is indicated by an increase in the lower pharyngeal airway size exceeding 15 mm, are supported by these observations. This was in accordance with a study done by Jain et al. [18] who also found similar results.

The pharyngeal structures and dentofacial pattern are expected to interact as a result of these findings, which show a strong relationship between the pharynx and dentofacial structures. This motivates orthodontic interest. As a result, the pharyngeal airway’s clinical relevance should not be undervalued, especially in adolescents in whom maxillary and mandibular growth and development are crucial.

The diagnosis of the developing class III malocclusion caused by the tongue’s forward posture also depends on this information. Visceral interferences, like increased lymphoid tissue, respiratory embarrassment, or a larger tongue, are a few of the potential causes of the tongue’s forward position. Another situation is when class II malocclusion is developing, the presence of narrow upper and lower pharyngeal airways can help in early diagnosis and better utilization of growth modification appliances to correct the malocclusion.

Therefore, this information may enable a trained physician to test patients for potential respiratory diseases at an early stage and start appropriate therapy at the relevant time to detect and prevent the onset of malocclusion at the right time [19].

Malkoc et al. determined that cephalometric films were highly reliable and reproducible for estimating pharyngeal airway dimensions [20].

As the study was conducted in a general population, these findings are applicable to other populations and can thus be extrapolated. Although the sample size in the current study was smaller, which could be a limitation. Therefore, further research is needed to correlate the palatal depth with the pharyngeal airway in all skeletal patterns with larger sample sizes.

## Conclusions

The palatal index of subjects with class II malocclusion had the highest mean value compared with class I and class III subjects. The mean value for the upper airway was found to be the greatest in class I as compared with class II and class III malocclusions. Moreover, the mean value for lower airway was found to be greatest in class III as compared to class I and class II malocclusions.
